# Observation of photo-induced plasmon–phonon coupling in PbTe via ultrafast x-ray scattering

**DOI:** 10.1063/4.0000133

**Published:** 2022-03-14

**Authors:** M. P. Jiang, S. Fahy, A. Hauber, É. D. Murray, I. Savić, C. Bray, J. N. Clark, T. Henighan, M. Kozina, A. M. Lindenberg, P. Zalden, M. Chollet, J. M. Glownia, M. C. Hoffmann, T. Sato, D. Zhu, O. Delaire, A. F. May, B. C. Sales, R. Merlin, M. Trigo, D. A. Reis

**Affiliations:** 1 Stanford PULSE Institute, SLAC National Accelerator Laboratory, Menlo Park, California 94025, USA; 2 Stanford Institute for Materials and Energy Sciences, SLAC National Accelerator Laboratory, Menlo Park, California 94025, USA; 3 Department of Physics, Stanford University, Stanford, California 94305, USA; 4 Tyndall National Institute and Department of Physics, University College, Cork, Ireland; 5 Departments of Physics and Materials, Imperial College London, London SW7 2AZ, United Kingdom; 6 Department of Applied Physics, Stanford University, Stanford, California 94305, USA; 7 Department of Materials Science and Engineering, Stanford University, Stanford, California 94305, USA; 8 Linac Coherent Light Source, SLAC National Accelerator Laboratory, Menlo Park, California 94025, USA; 9 Department of Mechanical Engineering and Materials Science, Duke University, Durham, North Carolina 27708, USA; 10 Materials Science and Technology Division, Oak Ridge National Laboratory, Oak Ridge, Tennessee 37831, USA; 11 Department of Physics, University of Michigan, Ann Arbor, Michigan 48109, USA

## Abstract

We report the observation of photo-induced plasmon–phonon coupled modes in the group IV–VI semiconductor PbTe using ultrafast x-ray diffuse scattering at the Linac Coherent Light Source. We measure the near-zone-center excited-state dispersion of the heavily screened longitudinal optical (LO) phonon branch as extracted from differential changes in x-ray diffuse scattering intensity following above bandgap photoexcitation. We suggest that upon photoexcitation, the LO phonon-plasmon coupled (LOPC) modes themselves become coupled to longitudinal acoustic modes that drive electron band shifts via acoustic deformation potentials and possibly to low-energy single-particle excitations within the plasma and that these couplings give rise to displacement-correlations that oscillate in time with a period given effectively by the heavily screened LOPC frequency.

In polar semiconductors, Fröhlich electron–phonon interactions lead to strong coupling between collective electronic and longitudinal lattice excitations. The coupling can be pronounced in the group IV–VI compounds due to the combination of high polarizability and large longitudinal optical (LO)/soft transverse optical (TO) phonon splitting near zone center. This leads to rapidly dispersing LO phonon–plasmon coupled (LOPC) modes^1–4^ that can affect nonequilibrium properties, such as carrier relaxation^5^ and transport,^6^ and have implications for thermoelectric transport,^7^ ferroelectricity,^8^ and superconductivity at high carrier densities.^9^ Inelastic neutron scattering (INS) measurements^1,2,10–12^ on several doped group IV–VI semiconductors, including PbTe, show an anomalous dip in the low-wavevector (long-wavelength) dispersion of the LO phonon branch, due to screening from the free carrier concentrations.

Ultrafast photoexcitation can be used to transiently control material properties, for example, through the excitation of large amplitude vibrational motion or the generation of large carrier densities. Such excitation can, furthermore, lead to a nonequilibrium state with dramatically different properties from the ground state. In the case of PbTe, where the LO–TO splitting is large, we may, therefore, expect that photoexcitation could be an effective control parameter for nonequilibrium properties. For example, the spectrum of THz emission from ultrafast laser excited PbTe was recently shown to be widely tunable depending on the photocarrier density.^13^ Thus, it is important to better understand the coupling between collective electronic and longitudinal lattice excitations. Photoexcited LO-phonon–plasmon coupled (LOPC) modes have been observed in all-optical experiments in the III–V compound GaAs^14–16^ as well as PbTe^17^ and PbTe_0.95_S_0.5_.^18^ Ultrafast, time-resolved x-ray diffuse scattering has the advantage that it allows us to directly observe the dispersion of the LOPC modes and their renormalization due to photo-excitation. Here, we present femtosecond Fourier-transform inelastic x-ray scattering (FT-IXS)^19–22^ measurements of near zone center excitations in photoexcited PbTe. Using this method, we have previously shown that near bandgap photoexcitation in PbTe couples the TO and transverse acoustic (TA) modes at high wavevector along the bonding direction, reducing the ferroelectric instability and stabilizing the paraelectric state.^22^ In this case, photoexcitation is also expected to strongly affect the LO phonon through coupling to the photoexcited plasma. Indeed, in the current work, we observe a heavily damped mode that strongly disperses with the increasing wavevector from near the zone center TO frequency to the LO frequency. We attribute the time- and wavevector-dependent signal to squeezed oscillations of the LOPC mode, likely due to coupling to longitudinal acoustic (LA) modes that drive shifts in the electronic bands via the acoustic deformation potentials.

The experiment was performed at the x-ray pump probe (XPP) instrument^23^ of the Linac Coherent Light Source (LCLS) x-ray free-electron laser. Details of the experimental setup can be found in Ref. 22. Briefly, infrared (IR) pulses of light (60 fs, 350 *μ*J, 0.6 eV) generated from an optical parametric amplifier laser were used as the pump source and hard x-ray pulses (50 fs, 8.7 keV) as the probing mechanism. The energy of the pump source was chosen to just exceed the direct bandgap of PbTe (∼0.31 eV at room temperature^24^). A large area Cornell-SLAC Pixel Array Detector (CSPAD) captured the resulting x-ray diffuse scattering over a wide region of reciprocal space. Diffuse scattering patterns were recorded at room temperature tracked as a function of time delay *τ* between the IR pump and x-ray probe pulses at binned step sizes of 100 fs.

We chose a fixed sample and detector configuration such that we capture scattering with a momentum transfer near the $\left(\bar{1}13\right)$ Brillouin zone. Brillouin zones with odd *h* +* k* + *l* are sensitive to both optical and acoustic phonons for the rock salt structure. The sample was detuned ∼1% from the Bragg condition for reciprocal lattice vector $G=(\bar{1}13)$ in reciprocal lattice units (rlu) to prevent the full intensity of the Bragg reflection from hitting the detector. In this geometry, two high-symmetry reduced wavevector directions are captured simultaneously (approximately): Γ to X with $q∼(0\;q_{y}\;0)$ and Γ to W with $q∼(q_{x}\;0\;q_{z}=2q_{x})$. Notably, in our measurement scheme, the two directions have varying sensitivity to phonon polarization due to the ${\left(Q\cdot e\right)}^{2}$ dependence in the scattering intensity, where **e** is the phonon polarization and Q=G+q is the momentum transfer (divided by ℏ). Thus, the diffuse scattering along Γ to X is primarily sensitive to phonons of transverse polarization [along the (001), *z*-direction]. Conversely, the diffuse scattering along Γ to W is largely sensitive to phonons of longitudinal polarization.

The differential scattering intensity $\delta I(\tau ;Q)=I(\tau ;Q)-I_{0}(Q)$ is collected as a function of time-delay, *τ*. $I_{0}(Q)$ is the unpumped signal, collected for time delays where the x-ray probe arrives prior to the pump pulse ( τ<0). The behavior along the two high-symmetry directions described above is shown in the time-domain in Fig. 1. Figures 1(a) and 1(b) show $\delta I(\tau ;Q)/I_{0}(Q)$ along the respective Γ to X and Γ to W wavevectors. Each trace represents the time-dependent differential changes in a pixel on the detector and, thus, a unique **Q** along one of the labeled high-symmetry wavevectors. The topmost traces depict data nearest to zone center. The traces extracted along the Γ to X wavevector reveal a strong decrease in scattering intensity immediately following the arrival of the pump pulse and weak modulations with long periods. The activity along Γ to X across the entire BZ has already been described in a prior report.^22^ Along the Γ to W wavevector, on the other hand, substantial coherent oscillations with much shorter periods are present, damping on a sub-picosecond timescale. Moreover, the oscillation period shortens with increasing **q** from Γ, as can easily be seen in the positive dispersion shown in the Fourier transform [Fig. 1(d)].

**FIG. 1.**
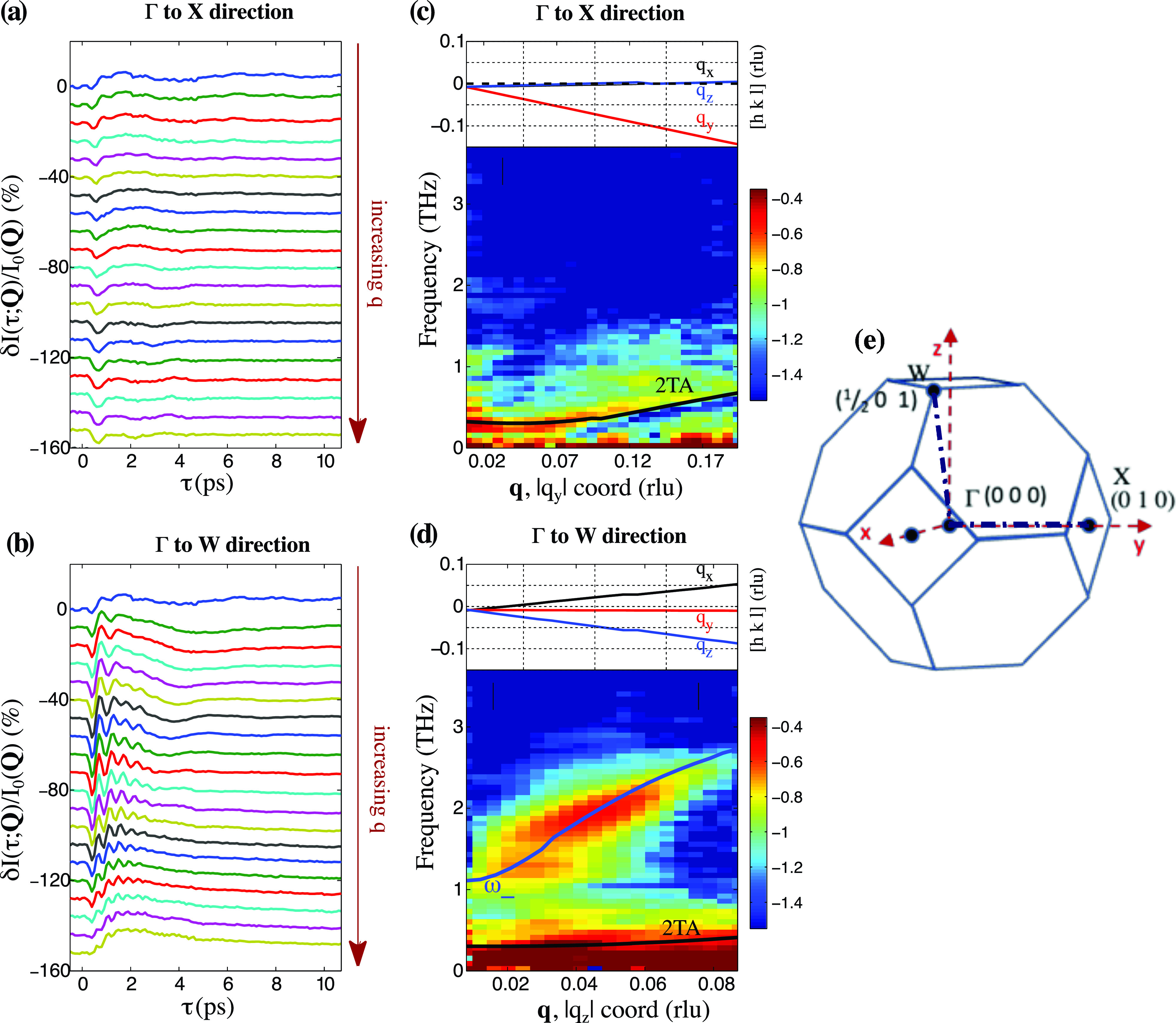
(a) and (b) Time-domain traces of relative differential diffuse scattering intensities extracted from pixels along the Γ to X and Γ to W wavevector directions, respectively, in the $\left(\bar{1}13\right)$ BZ. The topmost traces represent wavevector coordinates closest to Γ, whereas the bottommost traces represent coordinates furthest from Γ. (c) and (d) FT-IXS spectra along the (a) Γ to X and (b) Γ to W directions as extracted from the amplitudes following Fourier-transform analysis. The amplitudes are displayed on a base-10 logarithmic scale. The coordinate values $\left(q_{x}q_{y}q_{z}\right)$ for the reduced wavevector directions are plotted above the spectra. Black traces in the spectra are two-phonon dispersion frequencies from first-principles, constrained density functional theory (CDFT) calculations as detailed in Ref. 22. The blue trace in (d) is the calculated approximate trajectory of the screened LO phonon branch $\omega_{-}(q)$ in the long-wavelength limit as described in the text. (e) fcc Brillouin zone with representative X and W points.

A sudden change in the interatomic force constants before and after photoexcitation leads to the time evolution of the displacement correlations between phonon modes. The time-dependent diffuse x-ray signal is proportional to those displacement correlations and its Fourier transform gives the FT-IXS spectrum.^19,25^ We plot the FT-IXS spectra^19–21^ of the collected time-domain data in Figs. 1(c) and 1(d) for a range of **q** from near Γ toward X and W, respectively. The amplitudes are displayed in false color on a logarithmic (base-10) scale as a function of $\left|q_{y}\right|$ in Fig. 1(c) and $\left|q_{z}\right|$ for 1(d). The complete trajectories of **q** coordinate values for both wavevectors are displayed in rlu above the spectra.

We identify some of the features in the FT-IXS spectra with the help of first principles calculations^22,25–27^ to obtain the displacement correlations between phonon modes due to a sudden promotion of valence electrons to the conduction band. The full details of the calculations can be found in Ref. 22. In Fig. 1(c), an overtone transverse acoustic mode (2TA) is identified as shown by the black trace. The appearance of this mode near Γ is consistent with the results discussed in Ref. 22 in which the same mode is identified along Γ to X, all the way out to zone edge.

Similarly, along Γ to W, the calculated excited-state dispersion for the overtone TA mode is overlaid as a black trace in the spectrum of Fig. 1(d) and agrees well with the low-frequency feature near ∼0.25 THz. Note that although this specific wavevector direction is primarily sensitive to longitudinal phonon polarization, the overtone TA branch still appears due to a non-negligible residual sensitivity to transverse polarization. However, these calculations do not predict the intense and broad highly dispersive feature appearing at higher frequencies. As described below, we attribute this feature as likely due to the displacement correlations between the photoexcited LOPC mode and LA modes that also couple to the photoexcited plasma.

The interaction between plasmons and the LO phonon mode in polar materials^1–4^ screens the macroscopic electric field associated with the LO branch, reducing its strength by a factor equaling the low frequency dielectric constant $\varepsilon (q,0)=\varepsilon_{0}(1+k_{s}^{2}/q^{2})$, where *k_s_* is the Thomas–Fermi screening wavevector.^28^ In the long-wavelength limit, the coupling is largest when the zone center plasma frequency $\omega_{p}(q=0)$ equals the LO frequency $\omega_{LO}(q=0)$. Here, the plasma frequency depends on the concentration of free carriers *n*, their charge and effective mass *e* and $m^{*}$, the vacuum permittivity $\varepsilon_{0}$, and the high-frequency dielectric constant $\varepsilon_{\infty }$ as

$$\omega_{p}^{2}(q=0)=\frac{ne^{2}}{m^{*}\varepsilon_{0}\varepsilon_{\infty }}.$$ (1)

For group IV–VI semiconductors, $\omega_{p}(q=0)$ ∼ $\omega_{LO}(q=0)$ for carrier concentrations as small as ∼10^17^ cm^−34^.

At higher carrier densities, when $\omega_{p}(q=0)$ exceeds $\omega_{LO}(q=0)$, the dielectric function can be approximated in the quasistatic limit ( ω→ 0) as ε(q,0). For $q\ll k_{s}$, the electric field of the LO mode is dramatically screened leading to dispersion in the lower frequency coupled plasmon–LO phonon,^1^

$$\omega_{-}^{2}(q)=\omega_{TO}^{2}(q=0)+\frac{\omega_{LO}^{2}(q=0)-\omega_{TO}^{2}(q=0)}{\varepsilon (q,0)},$$ (2)

where $\omega_{TO}(q=0)$ is the zone center TO phonon frequency (∼0.95 THz for PbTe at room temperature).^11^

For large enough carrier density, such as in dense photoexcitation, the Fermi energy exceeds the thermal energy and we can approximate the carriers as a degenerate Fermi gas. In this case, we approximate the Fermi-wavevector within a single L-valley as $k_{F}={\left(3\pi^{2}(n/4)\right)}^{1/3}$, such that the Thomas–Fermi screening wavevector

$$k_{s}=\frac{e}{\hbar }{\left(\frac{3nm^{*3}}{{\left(\varepsilon_{0}\varepsilon_{\infty }\right)}^{3}\pi^{4}}\right)}^{1/6}.$$ (3)

Thus, in our experiments, strong plasmon–phonon coupling is expected from the initially low-carrier-density (4 × 10^17^ cm^−3^) n-type PbTe upon photoexcitation of $∼2\times 10^{20}{\;\text{cm}}^{-3}$ carriers per L-valley above the bandgap ( ∼0.5 % valence excitation). In this excitation regime, the frequency of the photoexcited plasma $\omega_{p}(q=0)$ (∼73 THz) far exceeds $\omega_{LO}(q=0)$ (∼3.42 THz for PbTe).^11^ Here, $k_{F}\approx 0.186$ rlu and $k_{s}\approx 0.083$ rlu, respectively. The use of a degenerate Fermi gas model to compute these parameters is reasonable since the approximate Fermi energy (∼1.3 eV) is larger that the expected thermal energy of the photoexcited carriers ( ≈0.5 eV). Moreover, the Debye screening wavevector for this temperature and density is very similar to the Thomas–Fermi screening wave vector.^29^ The particular details of this model do not have a significant impact on the forthcoming interpretation of our experimental results due to the weak $n^{1/6}$ dependence for the screening wavevector seen in Eq. (3).

The calculated dispersion in $\omega_{-}$ for this simple model at both equilibrium and photoexcited carrier density of $n=4\times 10^{17}{\text{cm}}^{-3}$ and $2\times 10^{20}{\text{cm}}^{-3}$ is shown in Fig. 2, purple and green traces, respectively. In the photoexcited case, *k_s_* is ∼2.7 times larger than that for the lower density even though the change in carrier density is more than three orders of magnitude, reflecting the $n^{1/6}$ dependence in Eq. (3). Nonetheless, a dramatic shift in the calculated equilibrium dispersion appears at low *q*. Due to the low carrier concentration of our samples, the expected equilibrium dispersion deviates appreciably from the LO frequency only in a small region near *q* = 0. This is reflective of the relatively weak screening of the LO phonon mode at such low carrier densities, and the screened portion of the dispersion would be difficult to resolve in most measurements. On the other hand, for the dense photoexcited case, the screened region of the dispersion of the LOPC mode extends considerably further from the zone center, reflective of the decreased screening length (increased *k_s_*).

**FIG. 2.**
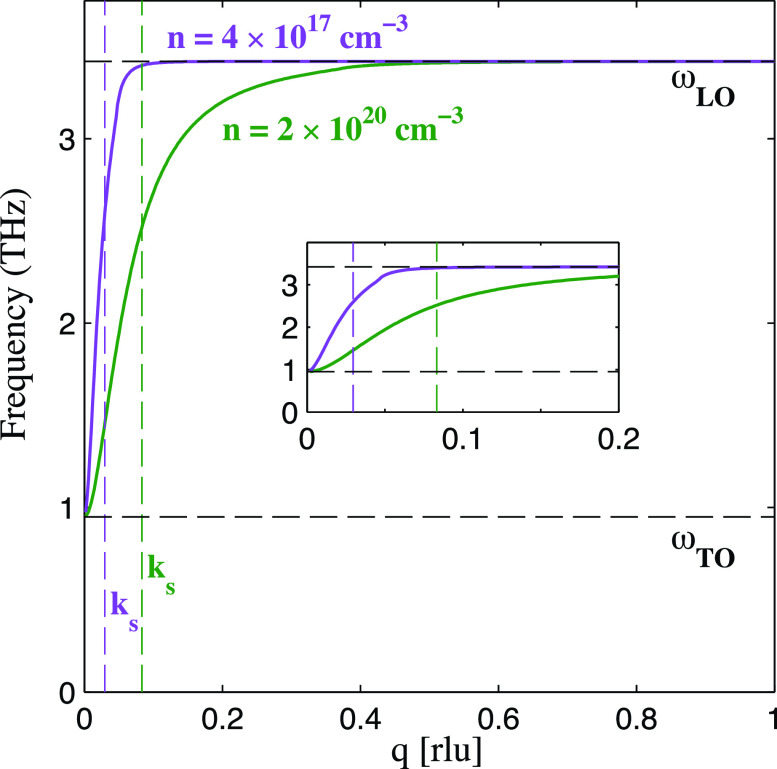
Model dispersion of the lower frequency LO phonon–plasmon coupled mode as a function of momentum (in rlu) under carrier densities of $n=2\times 10^{20}{\;\text{cm}}^{-3}$ (green line) and $n=4\times 10^{17}{\;\text{cm}}^{-3}$ (purple line). The higher carrier density matches the estimated photoexcited carrier density for this experiment, while the lower carrier density represents the initial concentration of the PbTe sample. The dashed black horizontal lines represent the zone center TO and LO frequencies of PbTe. The dashed colored vertical lines are the calculated Thomas–Fermi screening wavevectors. The inset figure plots the same dispersions in a shorter wavevector range in order to emphasize the difference.

The data in Fig. 1 extend to approximately the *k_s_* calculated above. Thus, we expect that the LOPC mode $\omega_{-}(q)$ should appear prominently in this region, given the strong sensitivity to longitudinal phonon polarization along this wavevector direction. The computed low-**q** screened LO phonon dispersion [Eq. (2)] along Γ to W is overlaid in blue. A close agreement with the highly dispersive spectral feature observed in the FT-IXS experiment is achieved for the estimated photoexcited carrier density. Thus, we attribute the broad high frequency dispersive feature to the photo-induced LOPC mode.

In the FT-IXS measurement, the LOPC mode would be expected to appear at either twice its frequency or in combination with low energy acoustic modes.^22^

The photoexcited electron–hole plasma gives rise to a coupling between the LO and LA modes. This arises because the shifts of the conduction and valence bands caused by strain act like an electric field on the carriers, driving them as the LA mode oscillates. The electric field of the charge carriers then interacts with the polar LO phonon. The details of this plasma-mediated interaction between the LO and LA modes are given in Appendix A. This LO–LA interaction, arising only in the presence of the photoexcited plasma, gives rise to a phonon squeezing signal at the sum and difference frequencies of the LO and LA modes, as observed in the experiment. The theory predicts that the signal goes to zero as q→0 and peaks at a wave vector, $q=k_{s}\varepsilon_{\infty }/\varepsilon_{\text{st}}$, where $\varepsilon_{\infty }$ is the high-frequency dielectric constant of PbTe (in the absence of the carriers) and $\varepsilon_{\text{st}}$ is the static dielectric constant of PbTe. This agrees well with observed range of wave vectors at which a large squeezing signal is detected. In Appendix B, we show that IXS oscillations at twice the LO mode frequency would not occur in the classical coupled phonon–plasma system, suggesting that such a signal should be weak in the current experiment. In principle, coupling of the LO mode to single-particle carried excitations could also give rise to IXS oscillations near the LO mode frequency.

The observed photo-induced plasmon–phonon state and the resulting screening of the LO phonon branch are consistent with early INS data on PbTe at degenerate carrier concentrations.^1,10,11^ The difference is that in the current experiment, we measure the screened LO mode dispersion in a material with much lower equilibrium carrier concentration ( $4\times 10^{17}$ cm^−3^ prior to photo-excitation) that is suddenly excited to very high concentration (10^20^–10^21^ cm^−3^). We note that from a plasma and screening perspective, our photoexcited density is in a similar regime as the highly doped crystals measured with INS; however, we emphasize that the current measurements do not reflect the spontaneous scattering from single LOPC and other excitation, but instead they represent the time-dependent scattering from the nonthermal and nonstationary state produced by the sudden excitation. This results in the measurement of correlated phonon pairs, for example, in the TA overtone and TA ± TO combination modes seen in Ref. 22 to span the entire Brillouin zone. Here, we have shown further that a high photoexcited carrier density in PbTe substantially affects the LO modes by sudden modification of the screening, giving rise to the generation of nonthermal LOPC modes of PbTe.

## Data Availability

The data that support the findings of this study are available from the corresponding author upon reasonable request.

## APPENDIX A: COUPLING OF THE CARRIER PLASMA TO ACOUSTIC PHONONS

The physical mechanism for this coupling is that the acoustic strain drives electrons because of the band shifts caused by the conduction band acoustic deformation potential:^7^ The longitudinal acoustic phonon gives rise to a local strain *s*(*x*) at the point *x*, where *x* measures the position along the wave direction, which then causes a shift of the conduction band energy,

$$\Delta E_{c}(x)=\frac{\partial E_{c}}{\partial s}\frac{\partial u_{a}}{\partial x}=\Xi_{c}\frac{\partial u_{a}}{\partial x},$$
(A1)

where $u_{a}(x)$ is the (longitudinal) displacement of the material at *x* due to the acoustic wave. As a term that affects the energy of electron carriers, this conduction band shift is identical to the effect of an external electrostatic potential,

$$V_{c}(x)=-\frac{\Xi_{c}}{e}\frac{\partial u_{a}}{\partial x},$$
(A2)

experienced by the electrons. The effective field determined by this potential drives the electron carriers, which in turn cause a (real) electric field that exerts a force on all charges, including the optical phonon. There is a similar effective electrostatic field, $V_{v}(x)=-(\Xi_{v}/e)\frac{\partial u_{a}}{\partial x}$, that drives the hole carriers. Writing the driving fields for electrons and holes in the following form:

$$\begin{array}{c} V_{c}=\frac{V_{c}+V_{v}}{2}+\frac{V_{c}-V_{v}}{2}\\ \text{and}\;\\ V_{v}=\frac{V_{c}+V_{v}}{2}-\frac{V_{c}-V_{v}}{2},\; \end{array}$$
(A3)

we can see that the second term $\pm (V_{c}-V_{v})/2$ drives electrons (negative charge) and holes (positive charge) identically, creating no change in the overall charge density and no change in the combined potential energy of the electrons and holes from the acoustic strain. Thus, for the purposes of our discussion here, we can use the average deformation potential, $\Xi =(\Xi_{c}+\Xi_{v})/2$ to give an effective, unscreened electrostatic potential for the plasma,

$$V_{a}=-\frac{\Xi }{e}\frac{\partial u_{a}}{\partial x},$$
(A4)

which drives both electrons and holes in the presence of acoustic strain.

### 1. Coupling of the LA and LO modes through the carrier plasma

To calculate the interaction energy between the strain field and the optical phonon, we will treat the electron–hole plasma as a dielectric medium with a wave vector and frequency-dependent dielectric constant ε(q,ω). Given the *q*-dependence of the dielectric function, it is convenient to transform all dynamical variables to their momentum representation with wave vector *q*. The potential energy density in a dielectric subjected to an external electric field, $D=\varepsilon_{0}\varepsilon_{\infty }\;E$, is

$$U=\frac{1}{2\varepsilon_{0}\varepsilon }D^{2}.$$
(A5)

In our problem, we need to change this expression slightly to take account of the fact that the potential due to the strain is not a true electrostatic potential; it is only experienced by the plasma, not the other charges (such as ions) in the system. If we write the effective field due to strain as

$$D_{a}=-\varepsilon_{0}\varepsilon_{\infty }\frac{\partial V_{a}}{\partial x}=\varepsilon_{0}\varepsilon_{\infty }\frac{\Xi }{e}\frac{\partial^{2}u_{a}}{\partial x^{2}}=-\varepsilon_{0}\varepsilon_{\infty }\;q^{2}\frac{\Xi }{e}u_{a}$$
(A6)

and *D_o_* is equal to the (bare, unscreened) electric displacement field due to the polar phonon, we can eliminate the unphysical terms in $D_{a}^{2}$ and $D_{a}D_{o}$ by subtracting their values when $\varepsilon =\varepsilon_{\infty }$, the high-frequency dielectric constant (i.e., when the electron–hole plasma is absent), leaving only the contributions from interaction of the acoustic strain and the LO phonon with the polarized plasma, along with the electrostatic interaction of the usual LO phonon electric field with itself. This gives a physical energy density in the strained system,

$$\begin{array}{c} U=\frac{1}{2\varepsilon_{0}\varepsilon }{\left[D_{a}+D_{o}\right]}^{2}-\frac{1}{2\varepsilon_{0}\varepsilon_{\infty }}\left\{{\left[D_{a}+D_{o}\right]}^{2}-D_{o}^{2}\right\}\\ =\frac{1}{2}\left\{\frac{1}{\varepsilon_{0}\varepsilon }-\frac{1}{\varepsilon_{0}\varepsilon_{\infty }}\right\}D_{a}[D_{a}+2D_{o}]+\frac{1}{2\varepsilon_{0}\varepsilon }D_{o}^{2}. \end{array}$$
(A7)

If *u_o_* is the optical phonon displacement, then the corresponding (bare) electric displacement field is

$$D_{o}=-\mathrm{ZeN}u_{o},$$
(A8)

where *Ze* is the Born effective charge, *N* is the number of crystal unit cells per unit volume, and *u_o_* is the LO phonon mode displacement. With these expressions for *D_a_* and *D_o_*, the energy of the fields is

$$\begin{array}{c} U=\frac{1}{2}\left\{\frac{\varepsilon_{\infty }}{\varepsilon }-1\right\}\frac{\Xi }{e}\frac{\partial^{2}u_{a}}{\partial x^{2}}\left(\varepsilon_{0}\varepsilon_{\infty }\frac{\Xi }{e}\frac{\partial^{2}u_{a}}{\partial x^{2}}-2ZeNu_{o}\right)+\frac{N}{2}\frac{{\left(Ze\right)}^{2}N}{\varepsilon_{0}\varepsilon }u_{o}^{2}\;, \end{array}$$
(A9)

or in momentum representation

$$\begin{array}{c} U=\frac{1}{2}\left\{\frac{\varepsilon_{\infty }}{\varepsilon }-1\right\}\frac{\Xi }{e}q^{2}u_{a}\left(\varepsilon_{0}\varepsilon_{\infty }\frac{\Xi }{e}q^{2}u_{a}+2ZeNu_{o}\right)+\frac{N}{2}\frac{{\left(Ze\right)}^{2}N}{\varepsilon_{0}\varepsilon }u_{o}^{2}. \end{array}$$
(A10)

The term

$$U_{a-o}=\left\{\frac{1}{\varepsilon }-\frac{1}{\varepsilon_{\infty }}\right\}\;\varepsilon_{\infty }\;\Xi ZN\;q^{2}u_{a}u_{o}$$
(A11)

gives the interaction between the LO and LA phonon, mediated by the photoexcited electron–hole plasma.

### 2. Equations of motion for LO and LA modes with carrier screening

In the coupled optical-acoustic phonon system, the total potential energy per unit cell is the sum of *U* with the acoustic strain energy and the energy associated with the local optical phonon restoring forces,

$$e=\frac{U}{N}+\frac{M}{2}v^{2}q^{2}|u_{a}{|}^{2}+\frac{\mu }{2}\omega_{T}^{2}u_{o}^{2},$$
(A12)

where *M* is the mass per unit cell, *μ* is the reduced mass per cell, *v* is the speed of sound, and *ω_T_* is the TO phonon frequency.

The kinetic energy per unit cell is

$$K=\frac{M}{2}|{\dot{u}}_{a}{|}^{2}+\frac{\mu }{2}|{\dot{u}}_{o}{|}^{2}\;.$$
(A13)

Scaling the field variables, *u_o_* and *u_a_*, to a standard form

$$z_{o}=\sqrt{\mu }u_{o}\;\text{and}\;z_{a}=\sqrt{M}u_{a},$$
(A14)

for which $K=|{\dot{z}}_{o}{|}^{2}+|{\dot{z}}_{a}{|}^{2}$, we can then write the coupled equations of motions for *z_o_* and *z_a_* in the standard form

$${\ddot{z}}_{o}=-\left[\omega_{T}^{2}+\frac{\varepsilon_{\infty }}{\varepsilon }\omega_{I}^{2}\right]z_{o}-C_{oa}\left\{\frac{1}{\varepsilon }-\frac{1}{\varepsilon_{\infty }}\right\}\omega_{I}^{2}\;z_{a},$$
(A15)

$${\ddot{z}}_{a}=-C_{oa}\left\{\frac{1}{\varepsilon }-\frac{1}{\varepsilon_{\infty }}\right\}\omega_{I}^{2}\;z_{o}-{\left(v^{\prime }\right)}^{2}q^{2}z_{a},$$
(A16)

where $\omega_{I}^{2}=\omega_{L}^{2}-\omega_{T}^{2}=\frac{Ne^{2}}{\mu \varepsilon_{0}\varepsilon_{\infty }}$ and

$$C_{oa}=\frac{\Xi \;\varepsilon_{\infty }}{ZNe^{2}}\sqrt{\frac{\mu }{M}}\;q^{2}$$
(A17)

and

$${\left(\frac{v^{\prime }}{v}\right)}^{2}=1+\frac{\varepsilon_{0}\varepsilon_{\infty }^{2}}{Ne^{2}}\left\{\frac{1}{\varepsilon }-\frac{1}{\varepsilon_{\infty }}\right\}\frac{\Xi^{2}}{Mv^{2}}q^{2}\;.$$
(A18)

### 3. Coupled LO–LA motion in the static screening approximation

Since the phonon frequencies are much lower than the plasma frequency for the photoexcited plasma, we will consider screening in the quasi-static limit, ω→0, using the Debye–Hückel or Thomas–Fermi screening approximation,^29^

$$\begin{array}{c} \frac{\varepsilon (q)}{\varepsilon_{\infty }}=1+\frac{k_{s}^{2}}{q^{2}}\Rightarrow \frac{\varepsilon_{\infty }}{\varepsilon }=\frac{q^{2}}{k_{s}^{2}+q^{2}}\;\\ \text{and}\\ \frac{\varepsilon_{\infty }}{\varepsilon }-1=\frac{k_{s}^{2}}{k_{s}^{2}+q^{2}}\;, \end{array}$$
(A19)

where the screening length is^29^

$$\begin{array}{c} l_{D}=\frac{1}{k_{s}}=\sqrt{\frac{kT\;\varepsilon_{0}\varepsilon_{\infty }}{2e^{2}n}}\;(\text{Debye})\\ \text{or}\\ \sqrt{\frac{kT_{F}\;\varepsilon_{0}\varepsilon_{\infty }}{3e^{2}n}}\;(\text{Thomas}-\text{Fermi})\;. \end{array}$$
(A20)

Here, *T* is the temperature of the electron–hole plasma, *T_F_* is the Fermi temperature, and *n* is the electron (or hole) carrier density. Similar results are obtained with the Debye–Hückel or Thomas–Fermi screening approximations since the temperature *T* of the hot, photoexcited plasma is not very different from its Fermi temperature *T_F_* in this experiment.

The equations of motion for the system then become

$${\ddot{z}}_{o}=-\left[\frac{k_{s}^{2}\omega_{T}^{2}+q^{2}\omega_{L}^{2}}{k_{s}^{2}+q^{2}}\right]z_{o}+\kappa \;\frac{q^{2}}{k_{s}^{2}+q^{2}}\;\omega_{I}^{2}\;z_{a},$$
(A21)

$${\ddot{z}}_{a}=\kappa \;\frac{q^{2}}{k_{s}^{2}+q^{2}}\;\omega_{I}^{2}\;z_{o}-v^{2}q^{2}\left[\frac{k_{s}^{2}+(1-\alpha )q^{2}}{k_{s}^{2}+q^{2}}\right]z_{a},$$
(A22)

where

$$\kappa =\frac{2}{Z}\frac{\Xi }{kT}\sqrt{\frac{\mu }{M}}\frac{n}{N}\;(\text{Debye})\text{or}\;\frac{3}{Z}\frac{\Xi }{kT_{F}}\sqrt{\frac{\mu }{M}}\frac{n}{N}\;(\text{Thomas}-\text{Fermi})$$
(A23)

is a dimensionless constant that characterizes the coupling strength between acoustic and optical modes induced by the electron–hole plasma and

$$\alpha =2\;\frac{\Xi }{kT}\;\frac{\Xi }{Mc^{2}}\;\frac{n}{N}\;(\text{Debye})\text{or}\;3\;\frac{\Xi }{kT_{F}}\;\frac{\Xi }{Mv^{2}}\;\frac{n}{N}\;(\text{Thomas}-\text{Fermi})$$
(A24)

is another dimensionless constant that determines softening of the acoustic modes.

In PbTe, the Born effective charge *Z* = 6.5,^11^ and the average deformation potential for conduction and valence bands, $\Xi =\xi_{d}/3+\;2\xi_{u}/3=4.3$ eV,^7,30^ $\sqrt{\mu /M}=0.485,\;v=1850$ m/s, *M* = 335 amu, $Mv^{2}=11.8$ eV. Typically, $kT_{F}\approx kT\approx 0.5-1$ eV for the photoexcited plasma. With 0.5% of the valence electrons excited to the condition band, n/N=0.05 since there are ten electrons in the valence bands. With these parameters,

κ≈0.05 and α≈0.2 .
(A25)

The small but non-negligible value of *κ* suggests that the weak coupling between the acoustic and LO modes, which grows from zero at *q* = 0, gives rise to the diffuse scattering oscillation. This agrees well with the experiment. However, the very broad, frequency-dependent anharmonic linewidth of the TO phonon (see below) obscures the splitting of the $\omega_{o}\pm \omega_{a}$ frequencies in the experiment.

Thus, defining screened optical and acoustic vibration frequencies, *ω_o_* and *ω_a_*, so that

$$\begin{array}{c} \omega_{o}^{2}=\frac{k_{s}^{2}\omega_{T}^{2}+q^{2}\omega_{L}^{2}}{k_{s}^{2}+q^{2}}\\ \text{and}\\ \;\omega_{a}^{2}=v^{2}q^{2}\frac{k_{s}^{2}+(1-\alpha )q^{2}}{k_{s}^{2}+q^{2}}, \end{array}$$
(A26)

the dynamical matrix is

$$D=\left[\begin{array}{cc} \omega_{o}^{2} & -\kappa \;\frac{q^{2}}{k_{s}^{2}+q^{2}}\;\omega_{I}^{2}\\ -\kappa \;\frac{q^{2}}{k_{s}^{2}+q^{2}}\;\omega_{I}^{2} & \omega_{a}^{2} \end{array}\right],$$
(A27)

which has eigenvalues

$$\omega_{\pm }^{2}=\frac{\omega_{o}^{2}+\omega_{a}^{2}}{2}\pm \sqrt{{\left(\frac{\omega_{o}^{2}-\omega_{a}^{2}}{2}\right)}^{2}+\kappa^{2}\;{\left[\frac{q^{2}}{k_{s}^{2}+q^{2}}\;\omega_{I}^{2}\right]}^{2}},$$
(A28)

and normal modes, $\left|\pm \rangle =\text{cos}\;\theta_{\pm }|o\rangle +\text{sin}\;\theta_{\pm }|a\right\rangle$, where |o⟩ and |a⟩ are the optic and acoustic modes, respectively, before excitation and

$$\text{tan}\;\theta_{\pm }=\kappa \;\frac{q^{2}}{k_{s}^{2}+q^{2}}\;\frac{\omega_{I}^{2}}{\omega_{\pm }^{2}-\omega_{a}^{2}}\;.$$
(A29)

[Note that $\text{cos}\;(\theta_{-})=-\text{sin}\;(\theta_{+})$ and $\text{sin}\;(\theta_{-})=\text{cos}\;(\theta_{+})$; we will refer to $\theta_{+}$ simply as *θ* in the remaining analysis.]

### 4. Squeezing signal at the LO–LA sum and difference frequency

Using the classical density matrix for the thermal state before excitation,^31^

$$\rho =kT_{\text{latt}}\left(\frac{\left|o\rangle \langle o\right|}{\omega_{T}^{2}}+\frac{\left|a\rangle \langle a\right|}{v^{2}q^{2}}\right),$$
(A30)

where $T_{latt}$ is the lattice temperature (not the electron–hole plasma temperature *T*), and the propagation of the normal modes after excitation, given by

$$|\pm (t)\rangle =|\pm \rangle \;\text{cos}\;(\omega_{\pm }t)e^{-\gamma_{\pm }t/2},$$
(A31)

where $\gamma_{\pm }$ is the amplitude decay rate of mode |±⟩, we find that the component of the squared optical mode displacement detected by x-ray diffuse scattering, which oscillates at frequency $\omega_{+}\pm \omega_{-}$, is

$$\begin{array}{c} {\left\langle u_{o}^{2}\right\rangle }^{\omega_{+}\pm \omega_{-}}=\frac{kT_{\text{latt}}}{2\mu }{\;\text{sin}\;}^{2}(2\theta )\left\{\frac{1}{\omega_{T}^{2}}-\frac{1}{v^{2}q^{2}}\right\}\\ \times \text{cos}\;(\omega_{+}t)\;\text{cos}\;(\omega_{-}t)e^{-(\gamma_{+}+\gamma_{-})t/2}\;. \end{array}$$
(A32)

For small values of *κ*,

$$\text{sin}\;\theta \approx \theta \approx \kappa \frac{\omega_{I}^{2}}{\omega_{o}^{2}-\omega_{a}^{2}}\frac{q^{2}}{k_{s}^{2}+q^{2}}\;.$$
(A33)

For the range of *q* considered here, $\omega_{T}^{2}\gg v^{2}q^{2}$ and the term $1/\omega_{T}^{2}-1/{\left(vq\right)}^{2}\approx -1/{\left(vq\right)}^{2}$, and since $\omega_{o}^{2}\gg \omega_{a}^{2}$, we can take $\omega_{o}^{2}-\omega_{a}^{2}\approx \omega_{o}^{2}$. The amplitude of each term in the diffuse scattering $\left\langle u_{o}^{2}\right\rangle$ that oscillates at frequency $\omega_{o}\pm \omega_{a}$ is then

$$\begin{array}{c} A_{\omega_{+}\pm \omega_{-}}=\frac{kT_{\text{latt}}}{\mu v^{2}}\frac{\theta^{2}}{q^{2}}\approx \kappa^{2}\;\frac{kT_{\text{latt}}}{\mu v^{2}}{\left(\frac{\omega_{I}}{\omega_{o}}\right)}^{4}\frac{q^{2}}{{\left[k_{s}^{2}+q^{2}\right]}^{2}}. \end{array}$$
(A34)

Substituting $\omega_{I}^{2}=\omega_{L}^{2}-\omega_{T}^{2}$, taking $\omega_{o}^{2}$ from Eq. (A26), and using the Lyddane–Sachs–Teller relation, the squeezing amplitude is

$$\begin{array}{c} A_{\omega_{+}\pm \omega_{-}}=\kappa^{2}\;\frac{kT_{\text{latt}}}{\mu v^{2}}{\left(1-\frac{\omega_{T}^{2}}{\omega_{L}^{2}}\right)}^{2}\frac{q^{2}}{{\left[{\left(\frac{k_{s}\omega_{T}}{\omega_{L}}\right)}^{2}+q^{2}\right]}^{2}}\\ =\kappa^{2}\;\frac{kT_{\text{latt}}}{\mu v^{2}}{\left(1-\frac{\varepsilon_{\infty }}{\varepsilon_{\text{st}}}\right)}^{2}\frac{q^{2}}{{\left[k_{s}^{2}\frac{\varepsilon_{\infty }}{\varepsilon_{\text{st}}}+q^{2}\right]}^{2}}\;, \end{array}$$
(A35)

where $\varepsilon_{\infty }$ is the high-frequency dielectric constant and $\varepsilon_{\text{st}}$ is the static dielectric constant. For PbTe, $\omega_{T}/\omega_{L}=0.278$^11^ and $\varepsilon_{\infty }/\varepsilon_{\text{st}}=0.077$. The amplitude vanishes at *q* = 0 and reaches a maximum value at $q=k_{s}\omega_{T}/\omega_{L}$, which would be the plasma screening wave vector if it were immersed in a medium with dielectric constant equal to the static value $\varepsilon_{\text{st}}$, rather than the high-frequency value $\varepsilon_{\infty }$. When the wave vectors *q* and *k_s_* are taken in reciprocal lattice units 2π/a, the squeezing amplitude can be written as a multiple of *a*^2^,

$$\frac{A_{\omega_{+}\pm \omega_{-}}}{a^{2}}=\frac{\kappa^{2}}{4\pi^{2}}\;\frac{kT_{\text{latt}}}{\mu v^{2}}{\left(1-\frac{\varepsilon_{\infty }}{\varepsilon_{\text{st}}}\right)}^{2}\frac{q_{\text{rlu}}^{2}}{{\left[k_{s,\text{rlu}}^{2}\frac{\varepsilon_{\infty }}{\varepsilon_{\text{st}}}+q_{\text{rlu}}^{2}\right]}^{2}},$$
(A36)

where $q_{\text{rlu}}=qa/2\pi$ and $k_{s,\text{rlu}}=k_{s}a/2\pi$. The maximum of $A_{\omega_{0}\pm \omega_{a}}$ for $q\approx k_{s}\varepsilon_{\infty }/\varepsilon_{\text{st}}$ is consistent with the observed intensity maximum of the squeezing signal.

### 5. Linewidth of squeezing oscillations

The linewidth of the optical mode for *q* near Γ, for which the local atomic motion is like the TO mode but at frequency *ω_o_*, is determined by the imaginary part of the anharmonic self-energy of the TO phonon at frequency *ω_o_*. The TO phonon self-energy has a very strong frequency dependence^22,32,33^ estimated from first-principles calculations^30^ to be very large ( $2.5\times 10^{12}$ s^−1^) at $\omega_{o}=\omega_{TO}\approx 1$ THz, reaching a maximum (10^13^ s^−1^) at $\omega_{o}=1.5$ THz, and falling to values less than 10^12^ s^−1^ as $\omega_{o}\to 3$ THz. This appears as a very broad peak in the squeezing spectrum,^22^ obscuring individual peaks at $\omega_{o}\;\pm \;\omega_{a}$ and particularly broad near $\omega_{o}\approx 1.5$ THz. The rate of dissipation of energy by currents in the plasma, $\sigma {\dot{P}}^{2}$, where *P* is the polarization of the plasma and $\sigma \approx ne^{2}\tau /m$ is the conductivity, is very small. Here, *τ* is the scattering time in the plasma, which we have found in photoexcited group-V semimetals to be a few femtoseconds. With the parameters for PbTe, the time constant for energy dissipation of the optic mode by Joule heating of the plasma is of the order of nanoseconds and contributes a negligible amount to the phonon linewidth.

## APPENDIX B: COUPLING OF CARRIER PLASMA OSCILLATIONS TO OPTICAL PHONONS

Here, we consider the classical dynamics of the LO phonon mode, coupled to the carrier plasma. In contrast to our analysis of the LO–LA coupling in Appendix A, we do not take account of the low-frequency part of the plasma response function, which describes electron–hole excitation and gives Thomas–Fermi or Debye screening. While neglecting these important screening effects, the analysis gives some insight into the absence of significant overtones at twice the LO phonon frequency in the diffuse scattering spectrum, in spite of a large change in LO mode frequency for small wave vectors.

Previously, we considered the plasma dynamics purely in terms of its static screening response to the LO mode electric field and the deformation potential of the LA mode; the dynamics of the plasma itself were not explicitly included as independent degrees of freedom. In a complementary view, the analysis in this section explicitly includes the classical plasma dynamics in a regime where screening by electron–hole excitations does not dominate the motion. In a further simplification, we do not consider the coupling of the LA phonon in this section.

### 1. Classical long-wavelength limit

As before, the kinetic energy (per unit volume) of the ions in the LO mode is $K_{I}=\mu N{\dot{u}}_{o}^{2}/2$, where *u_o_* is the displacement of the optic mode. The kinetic energy per unit volume of the plasma is $K_{p}=mn{\dot{u}}_{p}^{2}/2$, where *m* is the plasma reduced mass, *n* is the plasma number density, and *u_p_* is the displacement of the plasma. The potential energy of the ions (due to the local restoring forces) is $U_{o}=\omega_{T}^{2}N\mu u_{o}^{2}/2$, where *ω_T_* is the TO frequency. The potential energy density due to the electric field e (which is in turn due to the ionic and plasma polarization, *P_o_* and *P_p_*) is

$$U_{e}=\frac{1}{2\varepsilon_{0}\varepsilon_{\infty }}{\left[P_{o}+P_{p}\right]}^{2}=\frac{1}{2\varepsilon_{0}\varepsilon_{\infty }}{\left[\mathrm{NZe}u_{o}+neu_{p}\right]}^{2}.$$
(B1)

Including this with the potential energy density $N\mu \omega_{T}^{2}u_{o}$ due to local restoring forces, the equations of motion for the coupled LO–plasma oscillations are

$$N\mu {\ddot{u}}_{o}+N\mu \omega_{T}^{2}u_{o}+\frac{\mathrm{NZe}}{\varepsilon_{0}\varepsilon_{\infty }}\left[\mathrm{NZe}u_{o}+neu_{p}\right]=0$$
(B2)

and

$$nm{\ddot{u}}_{p}+\frac{ne}{\varepsilon_{0}\varepsilon_{\infty }}\left[\mathrm{NZe}u_{o}+neu_{p}\right]=0.$$
(B3)

Defining renormalized coordinates,

$$z_{o}=\sqrt{N\mu }\;u_{o}\;\text{and}\;z_{p}=\sqrt{nm}\;u_{p},$$

we can rewrite these equations of motion as

$${\ddot{z}}_{o}=-\left(\omega_{T}^{2}+\omega_{I}^{2}\right)z_{o}-\omega_{I}\omega_{p}\;z_{p}$$

and

$${\ddot{z}}_{p}=-\omega_{I}\omega_{p}\;z_{o}-\omega_{p}^{2}\;z_{p},$$

where, as defined previously, $\omega_{I}^{2}=\omega_{L}^{2}-\omega_{T}^{2}$. Thus, the dynamical matrix for the coupled system is

$$D=\left[\begin{array}{cc} \omega_{L}^{2} & \omega_{I}\omega_{p}\\ \omega_{I}\omega_{p} & \omega_{p}^{2} \end{array}\right],$$
(B4)

which has eigenvalues

$$\omega_{\pm }^{2}=\frac{\omega_{L}^{2}+\omega_{p}^{2}}{2}\pm \sqrt{{\left(\frac{\omega_{L}^{2}-\omega_{p}^{2}}{2}\right)}^{2}+\omega_{I}^{2}\omega_{p}^{2}},$$
(B5)

and eigenvectors $\left(\text{cos}\;\theta_{\pm },\;\text{sin}\;\theta_{\pm }\right)$, where

$$\text{tan}\;\theta_{\pm }=\frac{\omega_{\pm }^{2}-\omega_{L}^{2}}{\omega_{I}\omega_{p}}$$

and the inverse of *D* is

$$D^{-1}=\frac{1}{\omega_{T}^{2}}\left[\begin{array}{cc} 1 & -\omega_{I}/\omega_{p}\\ -\omega_{I}/\omega_{p} & \omega_{L}^{2}/\omega_{p}^{2} \end{array}\right].$$
(B6)

### 2. Ionic mean square displacement

In thermal equilibrium at temperature *T*, the equal-time, mean square correlations of *z_o_* and *z_p_* are

$$\left[\begin{array}{cc} \left\langle z_{o}^{2}\right\rangle & \left\langle z_{o}z_{p}\right\rangle \\ \left\langle z_{o}z_{p}\right\rangle & \left\langle z_{p}^{2}\right\rangle \end{array}\right]=kTD^{-1}=\frac{kT}{\omega_{T}^{2}}\left[\begin{array}{cc} 1 & -\omega_{I}/\omega_{p}\\ -\omega_{I}/\omega_{p} & \omega_{L}^{2}/\omega_{p}^{2} \end{array}\right].$$
(B7)

The mean square displacement of the ions,

$$\langle u_{o}^{2}\rangle =\frac{kT}{N\mu \;\omega_{T}^{2}},$$
(B8)

is related to the TO frequency only and does not depend on *ω_L_* or *ω_p_*, which seems to be a counterintuitive result.

The mean square ionic displacement is related to the mode eigenvectors and frequencies by

$$\frac{\left\langle z_{o}^{2}\right\rangle }{kT}=\frac{\;{\text{cos}}^{2}\;\theta_{+}}{\omega_{+}^{2}}+\frac{\;{\text{cos}}^{2}\;\theta_{-}}{\omega_{-}^{2}},$$
(B9)

where the first term on the r.h.s. is the contribution from the high-frequency mode and the second term is the contribution of the low-frequency mode. We note that $\theta_{-}=\theta_{+}+\pi /2$, so that $\text{cos}\;\theta_{-}=-\text{sin}\;\theta_{+}$. For example, when the plasma density is very low ( $\omega_{p}\ll \omega_{L}$), then the square-root in Eq. (B5) can be expanded by the binomial expansion to give $\omega_{-}\approx \omega_{p}\omega_{T}/\omega_{L}$ and $\omega_{+}\approx \omega_{L}$. In this regime, the angles, $\theta_{+}\approx 0$ and

$$\text{cos}\;\theta_{+}\approx 1\;\text{and}\;\;\text{cos}\;\theta_{-}=-\text{sin}\;\theta_{+}\approx -\frac{\omega_{I}\omega_{p}}{\omega_{L}^{2}}.$$
(B10)

Then, the contribution of the high-frequency mode to $\left\langle z_{o}^{2}\right\rangle$ is approximately $kT/\omega_{L}^{2}$ and the contribution of the low-frequency mode is

$$kT\frac{\;{\text{cos}}^{2}\;\theta_{-}}{\omega_{-}^{2}}=kT\frac{\;{\text{sin}}^{2}\;\theta_{+}}{\omega_{-}^{2}}\;\approx \;\frac{kT\omega_{I}^{2}}{\omega_{L}^{2}\omega_{T}^{2}}=\frac{kT}{\omega_{T}^{2}}-\frac{kT}{\omega_{L}^{2}}.$$
(B11)

Thus, although the low-frequency mode is primarily plasmon like in this case, its thermal mean square amplitude is sufficiently large (because of its low frequency) that it makes a significant contribution to the mean square displacement of the ions, even in the limit of vanishingly small plasma density. A similar analysis for high density, where the plasma frequency is much larger than the LO frequency, also gives this result. We emphasize that this analysis depends on the classical equipartition of energy and would not be exact in the quantum regime.

### 3. Photo-excitation of the electronic plasma

If the electronic plasma density is suddenly changed by photo-excitation from *n* to n′, the kinetic energy density *K_p_* and polarization *P_p_* of the plasma are initially unchanged, so that the renormalized electronic plasma variables are

$$\dot{z}\prime_{p}={\dot{z}}_{p}\;\text{and}\;z\prime_{p}=\sqrt{n/n\prime }\;z_{p}=(\omega_{p}/\omega \prime_{p})\;z_{p},$$
(B12)

immediately after the plasma density change. The ionic coordinates, *z_o_* and ${\dot{z}}_{o}$, are unchanged immediately after photo-excitation. This means that the correlations immediately after excitation are

$$\begin{array}{c} \left[\begin{array}{cc} \left\langle z_{o}^{2}\right\rangle & \left\langle z_{o}z\prime_{p}\right\rangle \\ \left\langle z\prime_{p}z_{o}\right\rangle & \left\langle {\left(z\prime_{p}\right)}^{2}\right\rangle \end{array}\right]=\left[\begin{array}{cc} \left\langle z_{o}^{2}\right\rangle & \left(\omega_{p}/\omega \prime_{p})\;\langle z_{o}z_{p}\right\rangle \\ \left(\omega_{p}/\omega \prime_{p})\;\langle z_{o}z_{p}\right\rangle & {\left(\omega_{p}/\omega_{p}^{\prime }\right)}^{2}\langle z_{p}^{2}\rangle \end{array}\right]\\ =\frac{kT}{\omega_{T}^{2}}\left[\begin{array}{cc} 1 & -\omega_{I}/\omega \prime_{p}\\ -\omega_{I}/\omega \prime_{p} & \omega_{L}^{2}/{\left(\omega_{p}^{\prime }\right)}^{2} \end{array}\right]=kT{\left[D^{\prime }\right]}^{-1}, \end{array}$$
(B13)

which are exactly at thermal equilibrium for the new plasma density. Therefore, *no squeezing oscillations (of u_o_ or u_p_) occur following the excitation process*.
